# Pyrogen retention: Comparison of the novel medium cut-off (MCO) membrane with other dialyser membranes

**DOI:** 10.1038/s41598-019-43161-2

**Published:** 2019-05-01

**Authors:** Michael Hulko, Verena Dietrich, Ilona Koch, Alexander Gekeler, Michael Gebert, Werner Beck, Bernd Krause

**Affiliations:** Baxter International Inc., Research & Development, Holger-Crafoord-Str. 26, 72379 Hechingen, Germany

**Keywords:** Nephrology, Renal replacement therapy

## Abstract

Haemodialysis effectively removes small solutes and smaller-sized middle molecules from the blood; however, the clearance of larger middle molecules, which have been associated with negative effects, is poor. The novel medium cut-off (MCO) dialysis membrane has larger pore sizes and a more open structure than other high-flux membranes, providing improved removal of larger middle molecules while retaining albumin. However, larger pore sizes may potentially increase permeability to pyrogens, including endotoxins and other bacterial contaminants, that could be present in the dialysis fluid. In this study, we tested the capacity of low-flux, high-flux, MCO and high cut-off dialyser membranes with different pore sizes to prevent pyrogens crossing from dialysate to the blood side in a closed-loop test system, differentiating among lipopolysaccharides, peptidoglycans and bacterial DNA using a toll-like receptor assay. Even though the bacterial contamination levels in our test system exceeded the acceptable pyrogen dose for standard dialysis fluid, levels of lipopolysaccharides, peptidoglycans and bacterial DNA in the blood-side samples were too low to identify potential differences in pyrogen permeability among the membranes. Our results suggest that MCO membranes are suitable for haemodialysis using ISO standard dialysis fluid quality, and retain endotoxins at a similar level as other membranes.

## Introduction

Microbial contamination of dialysis fluid can contribute to the pathological features of uraemia in patients receiving dialysis^1,2^. In particular, chronic exposure to low levels of microbial components such as endotoxins, peptidoglycans (PGs) and bacterial DNA (bDNA) can activate toll-like receptors (TLRs) in peripheral blood mononuclear cells, leading to the release of interleukin-1β, tumour necrosis factor and other proinflammatory cytokines^1,3^.

Endotoxins are a highly heterogeneous group of heat-stable lipopolysaccharides (LPS) that form the major component of the outer cell wall of Gram-negative bacteria^2,4,5^. The molecular weight of LPS ranges from 2 to more than 100 kDa, and larger aggregates known as micelles often form^1,2,5^. PGs are components of the bacterial cell wall of both Gram-positive and Gram-negative bacteria. They are formed of complex heteropolymers and have a molecular weight of more than 20 kDa^1,2,4,5^. bDNA contains a higher proportion of unmethylated CpG motifs than vertebrate genomic DNA and stimulates both innate and acquired immune responses^2^. The molecular weight of bDNA fragments is typically less than 5 kDa^1^.

To produce dialysis fluid, tap water first undergoes several stages of purification and filtration to remove 90–100% of inorganic and organic solutes, particulates, pyrogens and bacterial contaminants^1^. An excess of this purified water is then pumped to the patient’s individual dialysis station via a distribution loop. To avoid wastage, unused purified water from the distribution loop is recirculated^1^. Finally, the dialysis monitor generates dialysis fluid from purified water and fluid concentrates, and applies additional ultrafiltration steps^1^. Dialysis fluid prepared in this way is considered ‘ultrapure’ when it meets the ISO 11663:2014 standard of fewer than 0.1 colony-forming units/mL and fewer than 0.03 endotoxin units (EU)/mL, as determined using the *Limulus amebocyte* lysate (LAL) assay^6^.

There are several points at which bacterial contaminants can enter the dialysis system^1^. Microbial growth in the reverse osmosis (RO) module can compromise the permeability of the membranes of the water-purification system and allow pyrogens to enter the dialysis fluid. The interface between the individual dialysis stations and the distribution loop is another potential source of bacterial contamination that can pass back into the system, while stagnant water in the tubing can lead to the accumulation of biofilm and individual connectors may harbour bacteria.

While international standards exist to define the quality of water and dialysis fluid^6^, there are no agreed standards for testing the endotoxin retention of different dialysers. There are several factors that must be considered. An *in vitro* test should reflect the clinical situation in terms of fluid composition, type and concentration of contaminant, fluid circulation (particularly back filtration), membrane area and plasma exposure of the membrane. Inevitably, compromises must be made concerning the use of a defined source of contamination (typically isolated LPS from *Escherichia coli*) or a more clinically relevant mixture of contaminants derived from lysed bacteria (e.g. *Pseudomonas aeruginosa*), and with respect to the use of an adapted or miniaturized circulation system to account for the technical constraints of a laboratory environment. Crucially, specific assays are only available for some molecules and cell-based assays are not specific for certain molecule types. For instance, the LAL assay can only detect classical endotoxin, LPS and lipid A^7^, meaning that other potential bacterial components can go undetected. In the past, LAL assays varied in their sensitivity^7^, meaning that direct comparisons were often difficult to make. More sophisticated test systems are now available that detect not only a wider range of contaminants derived from Gram-positive and -negative bacteria, such as LPS, PGs and bDNA, but also mycoplasma, fungi and viruses. These assays exploit pattern-recognition receptors of the innate immune system to facilitate the detection of specific pathogens by recognizing highly conserved and class-specific molecules known as pathogen-associated molecular patterns^8^. Examples of these more versatile systems include the THP-1^9^ and TLR assays^8^.

Haemodialysis (HD) removes small solutes such as urea and creatinine and smaller-sized middle molecules from the blood, but the clearance of larger middle molecules (>15 kDa) is limited^10,11^. Haemodiafiltration (HDF) is an option to overcome the limitations of high-flux membrane permeability by creating convection through transmembrane pressure, thereby enhancing the removal of larger middle molecules^12^; however, HDF therapy is not suitable for, or available to, all patients. Furthermore, it can be costly and requires specialist equipment combined with high volumes of ultrapure dialysate and sterile substitution fluid. High cut-off (HCO) membranes remove larger toxins of up to 50 kDa during HD; however, they are reserved for acute applications such as the treatment of myeloma-related kidney disease^13^. The newly developed medium cut-off (MCO) membranes have an effective pore radius of 3.0–3.5 nm after contact with blood, allowing for the removal of an expanded range of uraemic toxins (up to 45 kDa) in comparison with conventional high-flux membranes in HD or HDF mode^14,15^.

The larger pore sizes of MCO membranes have raised concerns about the potential for increased membrane permeability to bacterial contaminants. Therefore, the aim of this work was to test the capacity of dialyser membranes with different pore sizes to prevent pyrogens crossing from the dialysate to the blood side, differentiating PGs, LPS and bDNA.

## Results

### Analysis of LPS by the LAL assay

Pore size was shown to not affect the retention capacity of different membranes for *E. coli* LPS (Table 1). On the basis of calculated logarithmic retention values (LRVs), there were no significant differences among the retention capacities of membranes with different pore sizes when tested with *E. coli* LPS (one-way analysis of variance, p = 0.110). When analysing LRVs for *P. aeruginosa* extracts, a significant difference between groups was observed (one-way analysis of variance, p < 0.001). Further analysis revealed a significant difference in the retention of LPS between MCO and low-flux membranes, MCO and high-flux membranes, and HCO and low-flux membranes (Holm–Sidak pairwise comparison, p < 0.001, p = 0.009, and 0.041 respectively [Table 2]), with a higher LRV observed for the MCO and HCO membrane in all comparisons, that is the MCO and HCO membranes were less permeable than the other membranes, respectively.

**Table 1:** LRVs for the different membranes tested, as determined by the LAL assay.

Membrane type	LPS (LAL *Escherichia coli*)			LPS (LAL *Pseudomonas aeruginosa*)		
	Dialysate (EU)	Blood (EU)	LRV	Dialysate (EU)	Blood (EU)	LRV
Low-flux	967 ± 309 [700–1400]	1.76 ± 0.92 [0.83–3.01]	2.78 ± 0.14	983 ± 240 [800–1400]	0.638 ± 0.527 [0.060–1.280]	3.37 ± 0.52
High-flux	867 ± 377 [600–1400]	0.39 ± 0.13 [0.25–0.56]	3.33 ± 0.23	1067 ± 344 [800–1500]	0.232 ± 0.132 [0.090–0.400]	3.71 ± 0.22
MCO	NS	NS	NS	783 ± 248 [400–1100]	0.028 ± 0.050 [0.005–0.130]	4.84 ± 0.50
High-flux with extended permeability	867 ± 236 [700–1200]	0.28 ± 0.01 [0.27–0.30]	3.47 ± 0.12	NS	NS	NS
HCO	733 ± 189 [600–1000]	0.41 ± 0.21 [0.11–0.61]	3.34 ± 0.43	750 ± 187 [400–900]	0.183 ± 0.338 [0.005–0.860]	4.25 ± 0.78

EU: endotoxin units; LAL, *Limulus amebocyte* lysate; LPS, lipopolysaccharides; LRV, logarithmic retention value; NS, not studied. Values are means ± s.d. [range].

**Table 2:** Pairwise comparison of LRVs for the different membranes tested, as determined by the LAL assay using *Pseudomonas aeruginosa* extract.

Comparison	Low-flux	High-flux	MCO
High-flux	p = 0.282		
MCO	p < 0.001*	p = 0.009*	
HCO	p = 0.041*	p = 0.191	p = 0.214

HCO, high cut-off; LAL, *Limulus amebocyte* lysate; LRV, logarithmic retention value; MCO, medium cut-off.

*Indicates a significant difference (Holm–Sidak pairwise comparison).

### Analysis of PG, LPS and bDNA by TLR assays

Pyrogenic signals were detected with the reporter cell lines TLR1/2, TLR2/6, TLR4/CD14 and TLR9 on the contaminated dialysate side of all tested membranes (Table 3). Little or, in most cases, no pyrogenic residue was detected with the reporter cell lines on the blood side of all tested membranes (Table 3). However, most test results were below the limit of detection of the assay and therefore a nominal value (0.001, the lowest reportable value of the photometric test system) was used to calculate the LRVs. This may have underestimated the LRVs as the true blood-side concentrations may be lower than 0.001; consequently, the true LRVs may be higher. Based on calculated LRVs, a significant difference in the retention capacity of membranes with different pore sizes could not be demonstrated for PG, LPS, or bDNA (Kruskal–Wallis one-way analysis of variance: TLR 1/2, p = 0.509; TLR 2/6, p = 0.590; TLR 4/CD14, p = 0.544; TLR 9, p = 0.191).

**Table 3:** LRVs for the different membranes tested, as determined by TLR assays.

Membrane type	PG (TLR1/2)			PG (TLR2/6)			LPS (TLR4/CD14)			bDNA (TLR9)		
	Dialysate (a.u.)	Blood (a.u.)	LRV	Dialysate (a.u.)	Blood (a.u.)	LRV	Dialysate (a.u.)	Blood (a.u.)	LRV	Dialysate (a.u.)	Blood (a.u.)	LRV
Low-flux	1.756 ± 0.241 [1.456–1.966]	0.001*	3.24 ± 0.06	1.676 ± 0.380 [1.265–2.024]	0.001*	3.21 ± 0.10	0.773 ± 0.122 [0.607–0.918]	0.002 ± 0.003 [0.001–0.010]	2.72 ± 0.495	0.171 ± 0.112 [0.036–0.266]	0.001*	2.11 ± 0.41
High-flux	1.795 ± 0.189 [1.578–1.966]	0.001*	3.25 ± 0.05	1.699 ± 0.378 [1.151–2.024]	0.001*	3.22 ± 0.10	0.763 ± 0.067 [0.648–0.809]	0.001*	2.88 ± 0.04	0.196 ± 0.092 [0.034–0.245]	0.001*	2.20 ± 0.38
MCO	1.655 ± 0.164 [1.388–1.782]	0.001 ± 0.001 [0.001–0.003]	3.14 ± 0.20	1.434 ± 0.172 [1.189–1.626]	0.001*	3.15 ± 0.05	0.740 ± 0.064 [0.655–0.809]	0.001*	2.87 ± 0.04	0.103 ± 0.075 [0.030–0.200]	0.001*	1.91 ± 0.35
HCO	1.805 ± 0.235 [1.418–1.966]	0.001*	3.25 ± 0.06	1.747 ± 0.318 [1.300–2.024]	0.001*	3.24 ± 0.08	0.810 ± 0.101 [0.654–0.936]	0.031 ± 0.038 [0.001–0.099]	1.89 ± 0.85	0.330 ± 0.096 [0.245–0.462]	0.030 ± 0.070 [0.001–0.172]	2.13 ± 0.93

a.u., absorbance units at 405 nm; bDNA, bacterial DNA; LPS, lipopolysaccharides; LRV, logarithmic retention value; PG, peptidoglycan; TLR, toll-like receptor. *Value below limit of detection and therefore presented as 0.001 to enable calculation of the LRV. Values are means ± s.d. [range].

## Discussion

This study did not detect differences in the retention capacities of polyarylethersulfone (PAES)-based three-layer membranes with different pore sizes for PG, LPS or bDNA as determined by the TLR assay. Even though the contamination level exceeded the acceptable pyrogen dose of standard dialysis fluid by a factor of nearly 10, the signals of PG, LPS and bDNA by the TLR-based reporter system in the blood side samples were mostly too low to identify potential differences in pyrogen permeability. No statistical difference in the retention of LPS by the different membranes were detected by the LAL assay when using *E. coli* LPS. Surprisingly, when using *P. aeruginosa* extract as the endotoxin source in the LAL assay a significant difference was observed in the retention capacity of the MCO membrane which was greater than that of the low-flux and high-flux membranes. This observation is contrary to the initial concerns that our study set out to investigate, namely that the larger pore size of the MCO membrane would increase permeability to pyrogens. If pore size was the driving factor then one would expect the LRV of the low-flux membrane to be highest, when, in most cases, it was the lowest. In all our experiments, the observed and largely similar levels of retention appeared to be irrespective of pore size and may be due to the intrinsic properties of the hydrophobic bulk polymer PAES (e.g. hydrophobic sites that provide retention), which is present in all the tested membranes. Furthermore, the intrinsic properties of endotoxins may also play a role; for example, cell-wall molecules (unlike soluble uraemic toxins) may have a propensity to aggregate and be retained. Further experiments are required to fully elucidate the mechanisms involved.

Many different contaminants are formed as a result of bacterial degradation and not all are detectable using the LAL assay. Nevertheless, LPS contamination of dialysis fluid of between up to 2 EU/mL and up to 7.68 EU/mL has been detected using the LAL assay^9,16^. A combined method using LAL and silkworm larvae plasma has detected PG levels of between 4.1 and 20 ng/mL in dialysis fluid from a central supply system^17^ and individual units^9^. As bDNA is only partially removed by ultrafiltration, it has been detected in HD fluid using bacterial transfer RNA-specific polymerase chain reaction (0.28 ± 0.02 μg/mL)^18^.

Our results, using two different reporter systems, indicate that pore size across a range of radii (3.1–10 nm) does not directly affect LPS, PG or bDNA retention in the studied dialysis membranes. To our knowledge, this study is the first to characterize the retention capacity of these membranes for PG and bDNA. Our *E. coli* LPS results agree with those of earlier experiments using a closed-loop system by Schindler *et al*., who demonstrated that the pore sizes of different Polyflux membranes did not significantly affect the transfer of LPS from dialysate to the blood side^19^.

While the small scale of closed-loop models has the advantage of allowing multiple tests under standardized conditions, such models are, by their nature, remote from the clinical situation. Clinical-simulation models offer an opportunity to test membranes under conditions that more closely represent membrane performance in the clinic. However, our results with a closed-loop system support the findings of a recent study using a clinical-simulation model. Using low-flux, high-flux, MCO and HCO membranes identical to those studied here, Schepers *et al*. tested endotoxin retention in a novel clinical model using *P. aeruginosa* and *Pelomonas saccharophila* extracts^20^. Using the LAL assay, they reported comparable *P. aeruginosa* LRVs to those obtained using our *in vitro* model, which were also comparable with the LRVs for *E. coli* LPS and did not differ significantly among the four types of membranes tested. The present study also found that PG and bDNA retention, as measured by the TLR reporter system, was similar among membranes with different pore sizes. Similarly, Schepers *et al*. also showed no significant difference in interleukin-1β expression of THP-1 cells, a measure of intact LPS, LPS fragments, PGs and short bDNA fragments, among low-flux, high-flux, MCO and HCO membranes^20^. Together, these studies suggest that in the clinical situation, these four membrane types would retain endotoxins from a range of bacteria and prevent them from passing into the patient.

Our findings should be considered in the context of the differences between soluble uraemic toxins (mostly proteins and small organic molecules) that pass across dialyser membranes and the chemically complex cell-wall–derived endotoxins that do not, as well as the associated difficulties of such mechanistic *in vitro* studies. The pyrogen retention properties of the investigated membranes may be explained by the formation of high molecular weight aggregates of pyrogen molecules and a mixed retention mechanism comprising size exclusion and adsorption; however, this study does not provide sufficient information to determine the precise mechanism. These results indicate that requirements and recommendations on dialysis fluid quality for conventional high-flux membranes should also apply to PAES/polyvinylpyrrolidone-based dialysis membranes with larger pores.

While our *in vitro* results, and the results of others^20^, suggest there is no difference in the retention of bacterial products among the different dialysis membranes tested here, there is a growing body of evidence offering preliminary support for a clinical benefit of membranes with larger pore sizes. A randomized open-label crossover trial recently compared MCO with high-flux dialysis over 4 weeks in 48 patients by assessing markers of inflammation^21^. The study demonstrated that MCO dialysers were able to modulate inflammation in patients undergoing chronic HD to a greater extent than high-flux dialysers^21^. While these trial results are promising, it should be noted that the studies reported to date have used surrogate markers of inflammation and there have, as yet, been no clinical investigations of clinical outcomes. As such, further studies are required.

One potential limitation of our study is that the MCO-Ci membrane was not available for testing during our experiments using the *P. aeruginosa* TLR and LAL assays. These experiments, which produced the majority of our data, were instead conducted using the MCO membrane. However, given that the *E. coli* LPS pyrogen load was far beyond that which would be observed in the clinic, and that the MCO-Ci membrane has larger pores, these data represent a worst-case scenario that may be extrapolated to the MCO membrane. Another potential limitation is the short duration of our simulated dialysis treatment (40 minutes versus ~4 hours in a real dialysis session). Given that the membranes were exposed to an almost ten times excess of pyrogen over 40 minutes, this can be extrapolated to an ~400-minute exposure within ISO limits, exceeding a typical dialysis session by over 2 hours.

In summary, while dialysis fluid quality in clinical practice is of importance and must be subject to quality-control measures as stipulated by standards, MCO membranes are suitable for delivering dialysis in HD mode using ISO-standard water and conventional dialysis fluid while retaining endotoxins, PGs and bDNA to a similar level as other, less open, membranes. MCO dialysers offer effective retention of bacterial products from conventional dialysis fluid without requiring an ultrapure water supply or other HDF infrastructure. Nevertheless, there may be other benefits of ultrapure water that are independent of the dialyser.

## Methods

### Membranes tested

The test items were mini-modules constructed using low-flux, high-flux, MCO or HCO membranes from commercial products (Polyflux 17L, Revaclear 400, Theranova 400 and Theralite 2100, respectively; Baxter, Hechingen, Germany) or the high-flux membrane with extended permeability taken from the investigational dialyser MCO-Ci (Baxter, Hechingen, Germany). All mini-modules had a membrane area of 360 cm^2^. The pore radii of the membranes, as determined by their dextran sieving characteristics^13^, and other characteristics are shown in Table 4.

**Table 4:** Characteristics of the tested membranes.

Device	Membrane type	Sterilization	Membrane polymer	Effective surface area (m^2^)	UF coefficient (mL/H/mmHg)	Pore radius* (nm)
Polyflux 17L	Low-flux	Steam	PAES/PA/PVP	1.7	12.5	3.1 ± 0.2
Revaclear 400	High-flux	Steam	PAES/PVP	1.8	54.0	3.9 ± 0.1
Theranova 400^†^	MCO	Steam	PAES/PVP	1.7	48.0	5.0 ± 0.1
MCO-Ci**	High flux with extended permeability	Steam	PAES/PVP	1.7	50.0	6.5 ± 0.2
Theralite 2100	HCO	Steam	PAES/PVP	2.1	52.0	10.0 ± 2.0

PA, polyamide; PAES, polyarylethersulfone; PVP, polyvinylpyrrolidone; UF, ultrafiltration. *Effective Stokes–Einstein radius: calculated from molecular-weight cut-off measured with polydisperse dextran. ^†^*Pseudomonas aeruginosa* TLR and LAL assay only. **Escherichia coli LAL assay only.

Our initial experiments conducted using the LAL assay in conjunction with *E. coli* O55:B5 endotoxin (see below) included the MCO-Ci high-flux membrane with extended permeability. For each type of membrane tested with *E. coli* O55:B5 endotoxin, three independent tests were performed. However, when we commenced the majority of our experiments using the TLR and LAL assays with the more clinically relevant *P. aeruginosa* extract (see below), the expiry date of the MCO-Ci membrane had been exceeded and we opted to use the similar, though not identical, MCO membrane in its place. For each type of membrane in each assay tested with *P. aeruginosa* extract, six independent tests were performed. Test articles which contained membrane leaks created by the preparation process were excluded.

### Sample generation

Assuming that a typical 1.8 m^2^ dialysis membrane is exposed to dialysis fluid with a maximum endotoxin load of 0.5 EU/mL (which represents the upper limit for conventional dialysis fluid meeting the ISO 11663:2014 standard^6^) for 300 min at a flow rate of 500 mL/min, the maximum exposure will be 4.2 EU/cm^2^. Assuming a worst-case scenario of 50 mL/min of back filtration in a dialyser with a 1.8 m^2^ membrane, the specific ultrafiltration rate will be 0.0028 mL/min/cm^2^. In the current study the *E. coli* and *P. aeruginosa* contaminant load, as determined by the LAL assay, was more than 500 EU/mL. This was delivered in 30 mL dialysis fluid and corresponded to at least 42 EU/cm^2^, or nearly 10 times the assumed maximum acceptable endotoxin load.

### Source of pyrogen for the LAL assay

Purified *E. coli* LPS are a high-potency source of LPS used as standards in the LAL assay and other endotoxin challenges^4^. Extracts derived from *P. aeruginosa* are not standardized with respect to the LAL assay, but offer a more clinically relevant source of contaminants^1^. Therefore, we opted to run our LAL assays with *E. coli* O55:B5 LPS (Lonza Walkersville Inc., Walkersville, MD, USA; product code 00193783) and extracts of *P. aeruginosa* separately.

### *P. aeruginosa* extract preparation

*P. aeruginosa* (DSMZ No. 50071, Leibniz Institute, Braunschweig, Germany) was pre-grown overnight in LB medium. This pre-culture was used to inoculate 1 L of LB medium and was further cultivated under constant shaking (37 °C; 160 rpm) until optical density at 600 nm was 1. The cells were then washed three times with phosphate-buffered saline (PBS, pH 7.4) to remove any residual medium using 20 mL/g ww (i.e. 20 mL of buffer per gram of pellet wet weight). After washing, the pellet was resuspended in PBS 1 mL/g ww and disrupted using high-pressure homogenization (Avestin B 15, Avestin, Mannheim, Germany; homogenization pressure of 20,000 psi for three rounds). The homogenate was clarified using centrifugation (13,000 × g; 30 min; 4 °C) and the supernatant (containing extracted endotoxins) was stored at −20 °C until required. The endotoxin content of one aliquot was determined using a LAL Kit (Kit# 88282 Pierce, Thermo Scientific, Waltham, MA, USA) to be 26,607,267 EU/mL.

### Pyrogen testing using a closed-loop system

**Figure 1:**
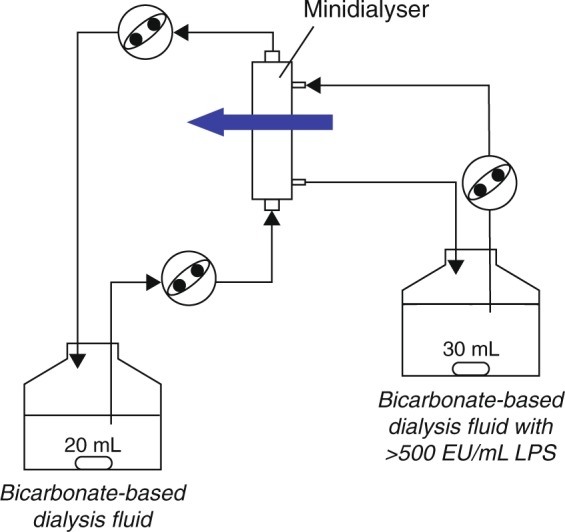
Closed-loop *in vitro* circuit simulating a haemodialysis treatment. Extracts of *P. aeruginosa* or *E. coli* O55:B5 LPS were added to the dialysate side at approximately 10 times the acceptable pyrogen dose of standard dialysis fluid, as determined by LAL assay. After 20 min of recirculation only and 20 min of recirculation with ultrafiltration of 16% of the blood-side flow rate from dialysate to the blood side (equivalent to an ultrafiltration rate of 1 mL/min/360 cm^2^), pyrogen activity on the blood and dialysate sides was measured.

Pyrogen retention was tested in a closed-loop *in vitro* circuit simulating an HD treatment at 37 °C (Fig. 1). Membranes were exposed to human plasma for 40 min and then intensively rinsed. The circuit was then filled with bicarbonate-based dialysis fluid prepared from concentrates A-Component D227 and B-Component D200 (MTN Neubrandenburg GmbH, Neubrandenburg, Germany). Extracts of *P. aeruginosa* or *E. coli* O55:B5 LPS were added to the dialysate side at approximately 10 times the acceptable pyrogen dose of standard dialysis fluid, as determined by LAL assay. After 20 min of recirculation only and 20 min of recirculation with ultrafiltration of 16% of the blood-side flow rate from dialysate to the blood side (equivalent to an ultrafiltration rate of 1 mL/min/360 cm^2^), pyrogen activity on the blood and dialysate sides was measured.

### Analysis of LPS by LAL assay

LPS concentrations were measured on the blood and dialysate sides using a chromogenic kinetic LAL assay (F4762E; Charles River Laboratories International, Inc., Wilmington, MA, USA) according to the manufacturer’s instructions. The assay was calibrated using standard endotoxin (CSE:EM34772; Charles River Laboratories International, Inc.).

### Analysis of PG, LPS and bDNA by TLR assay

All tests were conducted at the Fraunhofer Institute for Interfacial Engineering and Biotechnology, Stuttgart, Germany. Pyrogen activity was measured using recombinant TLR reporter cell lines with selectivity for PG by TLR1/2 and TLR2/6, for LPS by TLR4/CD14 and for bDNA by TLR9^8^.

A reporter plasmid-only cell line was used as a negative control to measure the background alkaline phosphatase expression of the reporter system and cell-culture medium was used as a reference item. The tumour necrosis factor receptor is capable of inducing nuclear factor-κB activation in NIH3T3 cells in the absence of pattern-recognition receptors^22^ and was used as a reference item for the control cell line. As positive controls, cell lines were induced by reference items as follows: TLR1/2 and TLR2/6, synthetic ligand, Pam, CysSK4^23^; TLR4/CD14, LPS^8^; TLR9, CpG oligodeoxynucleotide, ODN2006^24^.

### Cultivation of reporter cell lines

For each assay, a vial of the master cell bank was revitalized and seeded in a standard cell-culture flask (75 cm^2^ T-Flask) on day 1. Cells were cultured in 20 mL culture medium (DMEM supplemented with 10% foetal calf serum, 50 U/mL penicillin, 0.05 mg/mL streptomycin and 2 mmol/L l-glutamine [all Invitrogen, Carlsbad, CA, USA]). All cells were cultured at 37 °C in a 5% CO_2_ humidified atmosphere.

For the pathogen-associated molecular pattern assay, cells were seeded onto a 96-well plate at a density of 0.3 × 10^5^ cells/well in a final volume of 100 mL culture medium. To ensure equal culture conditions, culture plates were placed side by side and cultivated simultaneously at 37 °C in a 5% CO_2_ humidified atmosphere. After a cultivation period of 25 h, the medium was removed and replaced with fresh medium (DMEM, 0.5% foetal calf serum) to a final volume of 100 μL/well. Induction was performed for 18 h at 37 °C and 5% CO_2_.

### Detection

To determine the final secreted alkaline phosphatase value, 50 μL of the supernatant was transferred to a new 96-well plate (Greiner-F-plate, Greiner Bio-One, Kremsmünster, Austria) and 50 μL of substrate (*p*-nitrophenyl phosphate) was added. Activity of the reporter enzymes was measured photometrically (at 405 nm) using a UV-VIS reader (SpectraMax Plus 384, Molecular Devices GmbH, Ismaning, Germany) and data were recorded using SoftMax Pro version 5.01 (Molecular Devices, Sunnyvale, USA).

The decadic LRV of the ratio of dialysate and blood-side reporter system activity was taken as a measure of retention capacity.

### Statistical analysis

Per standard practice, no sample size calculations were applied to this laboratory investigation. Samples were generated in three independent experiments using *E. coli* LPS and six independent experiments using *P. aeruginosa* extract. Single measurements were performed for the LAL assays. TLR assays were repeated four times and the mean value (±s.d. and range) is reported. Statistical analysis was conducted using SigmaPlot version 12.5 (Systat Software Inc., San Jose, USA). The normality of data distribution was tested with the Shapiro–Wilk test. For normally distributed data, the one-way analysis of variance was used to detect differences between groups. Pairwise comparisons were then assessed with the Holm–Sidak method. For non-normally distributed data, the Kruskal–Wallis one-way analysis of variance on ranks was used to assess between-group differences. All tests were conducted with a significance level of p < 0.05.

## Data Availability

The datasets generated during and/or analysed during the current study are available from the corresponding author on reasonable request.
